# 

**Published:** 1904-11-15

**Authors:** 


### CONTENTS.

Compressed Air in Dentistry,

*By George Zederbaum, D.D.S.* 541

Means and Methods for Obtunding Dentine,
*By F. C. Collins, D.D.S.* 548

Cavity Preparation for Porcelain Fillings,
*By W. T. Reeves, D.D.S.* 555

Extension for Prevention: A Study of Conditions in the Mouth, - *By Frederick B. Noyes, B.A., D.D.S.* 564

National Association of Dental Examiners—Report of Committee on Colleges - - - - - - 575

EDITORIAL, - - - - - - - - 577

BIBLIOGRAPHY, - - - - - - - - 579

ANNOUNCEMENTS, - - - - - - - 580

OBITUARY, - - - - - - - - - 582

INDENTS, - - - - - - - - - 583

